# A Novel Transaortic Pulsatile Pump to Unload the Left Ventricle

**DOI:** 10.1016/j.jacbts.2025.01.012

**Published:** 2025-02-11

**Authors:** James Kim, Paula Rambarat, Allison Levin, Phillip C. Yang, Jeffrey Teuteberg, Arnold Seto, Morton Kern, Barry Greenberg, Manesh Patel, Marat Fudim

**Affiliations:** a Heart and Wellness Center, San Diego, California, USA; b Duke University School of Medicine, Durham, North Carolina, USA; c Stanford University School of Medicine, Palo Alto, California, USA; d Long Beach VA Health Care System, Long Beach, California, USA; e University of California, San Diego, California, USA

Temporary mechanical circulatory support (tMCS) systems have evolved over the past 40 years to match growing clinical needs of patients with advanced heart failure (HF) and cardiogenic shock. Contemporary tMCS devices include the pulsatile intra-aortic balloon pump; high-speed, continuous flow, rotor-based, microaxial flow pumps; and centrifugal flow pumps (TandemHeart, CentriMag; venoarterial extracorporeal membrane oxygenation). Despite significant evolution in tMCS design, many studies have failed to demonstrate significant benefits of these devices in patients with cardiogenic shock, and multiple observational studies have suggested harm with routine use of Impella CP in cardiogenic shock. Similarly, routine use of intra-aortic balloon pump and venoarterial extracorporeal membrane oxygenation in cardiogenic shock have also failed to show significant mortality benefit.

However, in 2024, the DanGer Shock (Danish-German Cardiogenic Shock) Trial demonstrated that routine implantation of Impella CP in addition to standard care alone is superior to standard care alone in reducing 6-month mortality among patients presenting with ST-segment elevation myocardial infarction and cardiogenic shock (45.8% vs 58.5%, HR: 0.74; 95% CI: 0.55-0.99; *P =* 0.04) in the intention to treat analysis.^1^ Importantly, the mortality benefit in the “as treated” analysis was not significant, and those patients randomized to Impella CP therapy had a higher risk of complications, eg, renal replacement therapy, sepsis, and limb ischemia. These results suggest that despite the hemodynamic benefit of continuous flow devices, there are often serious complications of high rotor speeds and transvalvular placement, including hemolysis, bleeding, arrhythmias, and in some cases death, that reduce their overall net benefit.

Novel axial flow pumps, which are designed to be placed in the aorta, rather than across the aortic valve, are being studied for use in HF. Devices such as Procyrion Aortix, Puzzle Medical ModulHeart, Second Heart Assist, and Reitan Catheter Pump have demonstrated promising data with improved renal perfusion, urine output, and improved hemodynamics.^2^^,^^3^ Beyond mitigating the risks of the transvalvular placement of microaxial flow pumps, these devices may provide safer options for use in mechanical aortic valves, severe aortic valvular disease, nondilated left ventricles, or left ventricular thrombus.

**Figure 1:**
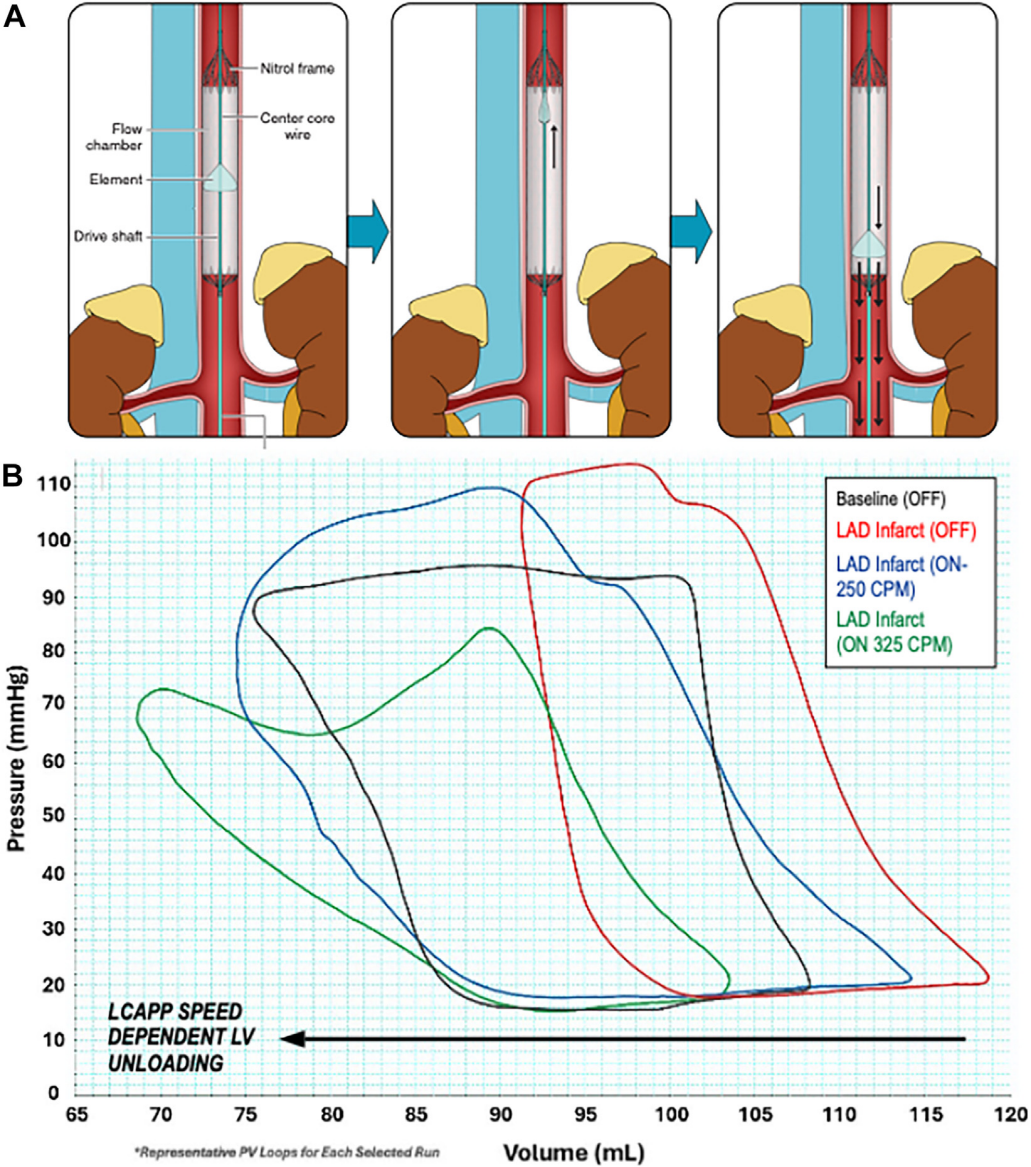
LCAPP and PV Loops (A) The linear cardiac assist pulsatile pump (LCAPP) is inserted via transfemoral 14-F sheath into the descending aorta via fluoroscopy. During the pump’s power stroke, blood is expelled forward creating pulsatile flow. (B) Pressure volume loop in 20-minute LCAPP study post-acute myocardial infarction at increasing motor speeds. LAD = left anterior descending artery; LV = left ventricular.

The Linear Cardiac Assist Pulsatile Pump (LCAPP) (Summacor is a novel, pulsatile pump currently able to deliver 7 L/min of flow at 325 cycles/min placed via a transfemoral 14-F sheath and positioned in the descending aorta. The LCAPP generates flow by way of an external reciprocating linear motor attached to the element (similar in shape to an umbrella), which opens to pull blood downstream on the power stroke, then collapses and returns to its prior position on the back stroke (Figure 1A). The intravascular portion of the device is self-expanding, and is made entirely of PTFE-coated nitinol components. Although confirmatory analyses measuring plasma-free hemoglobin and von Willebrand factors are still pending, Computational fluid dynamics studies suggest that the pump operates below the threshold for hemolysis (Hemolysis Index < 0.1) across all simulations.

The hemodynamic performance of the LCAPP was validated in a cohort of 9 pigs, with studies conducted on 6 of these animals using a 15-mm LCAPP, encompassing 2 healthy, 1 HF, and 3 acute myocardial infarction (AMI) pigs.^4^ Subsequently, the second-generation 20-mm LCAPP was implanted in 3 pigs and AMI models were induced. Pressure-volume loop catheters were placed to better define the mechanism of support. The AMI model was generated via left anterior descending artery ligation, and the HF model by acute beta-blocker and intravenous fluid loading. Arterial blood pressure was monitored via carotid and femoral arterial lines. A transaortic gradient was calculated and defined as the difference in femoral and carotid mean arterial pressure (MAP). Devices were removed at the end of the hemodynamic evaluation.

All devices were inserted and operated without complication or device malfunction. During support, femoral MAP and transaortic gradient significantly increased and left ventricular end-systolic pressure and left ventricular end-diastolic pressure (LVEDP) significantly decreased in all pigs. In the AMI cohort, which included 3 of the 15-mm and 3 of the 20-mm LCAPPs, the femoral MAP increased from a median of 77 mm Hg (Q1-Q3: 57-88 mm Hg) to 90 mm Hg (Q1-Q3: 73-109 mm Hg; *P* < 0.05), the LVEDP decreased from 20 mm Hg (Q1-Q3: 16-20 mm Hg) to 15 mm Hg (13-17 mm Hg; *P* < 0.05), and ST-segment elevations normalized in 2 animals during device support and recurred with cessation of support. Pressure-volume loops demonstrated consistent pressure and volume unloading (Figure 1B). In the pig with HF, after 1 hour of pump support without diuretic agents, decreases in central venous pressure (31 to 15 mm Hg) and LVEDP (25 to 11 mm Hg) were seen. Additionally, 1,600 mL of urine was produced over 2 hours with no evidence of hemoglobinuria.

In summary, the ongoing evolution of tMCS has been necessary to match the clinical needs of the advanced HF population. Most current tMCS devices can enhance flow considerably by using high-speed continuous flow rotors, but with costs related to hemocompatibility, reduced pulsatility, and the necessity of crossing the aortic valve. We propose a more efficient, pulsatile, positive displacement pump positioned in the descending aorta to unload the left ventricle in the setting of high-risk PCI, advanced HF, and cardiogenic shock. Although our preclinical data has been promising to date, we intend to continue studying the LCAPP’s effect on hemolysis and Von Willebrand factor multimers with the expectation that physiological pulsatility will naturally be more hemocompatible. Preliminary data has demonstrated improved MAP and lower filling pressures without evidence of hemolysis or device malfunction. As we move forward, the integration of novel tMCS technologies like the LCAPP into clinical practice holds transformative potential, but we await clinical trials to validate these promising findings, with the ultimate goal of expanding therapeutic options and improving outcomes for this complex patient population.
